# Supplementary material for: Are carbon emissions trading and green financial instruments synergistic? -Comprehensive quantitative research based on content analysis
Source: PLoS One. 2024 Mar 7;19(3):e0298601. doi: 10.1371/journal.pone.0298601 (PMC10919601; doi:10.1371/journal.pone.0298601)
Supplement: S1 Data — (ZIP) [file pone.0298601.s001.zip › Data/地级市Z_F工作报告.zip!/─■╧─/╬Γ╓╥╩╨/└┤╫╘╣1⁄2╓┌║┼í╛┬φ┐╦╩2╛▌═°í┐_2012.docx]

各位代表：

现在，我代表市人民政府向大会作政府工作报告，请予审议，并请市政协委员和其他列席人员提出意见。

实现逆势突破的2011年

刚刚过去的一年，是我市改革开放和现代化建设非常关键的一年。面对宏观经济环境新变化和我市改革发展新任务，在自治区党委、政府和市委的正确领导下，全市上下紧紧围绕科学发展、跨越发展这个主题，努力做好保增长、转方式、惠民生、促和谐各项工作，全面完成了市三届人大四次会议确定的各项目标任务，实现了“十二五”良好开局。

——经济增长好于预期。预计全年完成地区生产总值260亿元，增长13%，比全区平均增速高1个百分点。地方财政收入42.1亿元，增长28.6%，其中一般预算收入21.2亿元，增长35.1%。完成固定资产投资260亿元，增长42.8%，增幅居全区前列。全市地方财政收入、固定资产投资累计总额提前一年完成本届政府预期目标。

——两大任务再创佳绩。实施招商引资项目422个，到位资金279亿元，增长38.7%。落实上争项目2154个，落实批复资金68.5亿元，增长30%。

——居民收入持续提高。预计城镇居民人均可支配收入15580元，增长12.5%。农民人均纯收入5600元，增长11%。城镇新增就业1.5万人，登记失业率控制在4.25%以内。农村劳动力转移就业24.2万人次，实现劳务收入16.4亿元，分别增长20%和19%。

——对外开放取得实效。成功举办第四届中国（宁夏）回商大会等系列活动，配合自治区成功举办中华黄河坛落成典礼相关活动。与新疆吐鲁番地区、内蒙古阿拉善盟结为友好城市，疆煤进吴、联合办园取得积极进展。

——县域经济亮点频显。利通区农民人均纯收入位居全区前列，增速全市第一。红寺堡区创建了全国第一个慈善工业园。青铜峡市成为全区第二个国家级卫生城市。盐池县地方财政收入突破3亿元，综合实力领跑全区山区县。同心县固定资产投资增速位居全区前列。

这些成就，是在异常复杂的形势下取得的，全市上下为之付出了艰辛努力。一年来，我们紧紧围绕转变发展方式这条主线，主要做了以下工作：

（一）抓优化升级，为转变发展方式提供了产业支撑。着力优化经济结构，突出又好又快发展，三次产业分别增长6.2%、17.6%和8.7%，产业之比调整为16:53:31，更趋合理。

　　工业经济逆势前进。针对年初工业经济下行的严峻形势，及时调整政策，强化运行调度，较快扭转了不利局面，实现逐月回升。实施千万元以上重点项目100个，完成投资136亿元。锦辉长城年产300套风机塔筒等95个重点项目开工建设，宁夏嘉泽10万千瓦风电等30个项目基本建成，伊利年产19万吨液态奶等32个项目投产运行。“两城五园”完成基础设施投资4.2亿元，实施工业项目89个，新入园规模以上企业43家，园区承载能力和集聚效应进一步增强。预计全年规模以上工业企业完成增加值97亿元，增长16%。其中，地方工业比重达到51%，为实现工业经济增长目标发挥了重大作用。

　　农业经济稳中有进。农业“十大基地”建设规模稳步提升，全市种植优质粮食面积100万亩，粮食总产达93.8万吨，实现“八连增”。新建规模化奶牛牧场22个，建成自治区级现代农业示范基地16个、市级31个，新建大型供港蔬菜基地4个，金银滩奶牛核心区被农业部命名为国家级农业产业化示范基地。新培育市级以上农业产业化龙头企业42家、农民专业合作组织35家，累计分别达到133家和475家，流转土地66万亩。同心圆枣、盐池甘草、老苗月饼荣获中国驰名商标，“法福来”雪花面粉等11个农产品荣获全国农产品金奖。大力开展农田水利基本建设，全市共投入资金13.3亿元，改善灌溉面积45万亩，新增节水灌溉面积40万亩，集中整治农田100万亩，在全区“黄河杯”评比中连续第四年取得好成绩。预计全年完成农业总产值83亿元，增长6.8%。

第三产业加速前进。预计全年实现社会消费品零售总额60.3亿元，增长17.7%。利通区“十大市场”、“三大汽车服务区”，盐池汽车城、同心清水湾综合农贸市场等市场建设效果显现。大力推进“家电下乡”和“家电以旧换新”工程，累计销售家电产品23万台(件)，实现销售额5.5亿元。“万绨旎”民族服饰在沙特吉达设立了展销窗口，涝河桥牛羊肉被指定为世界大运会、全国民运会专供食品，预计全年实现产值115亿元，增长15%。旅游业重点建设项目全面加快，中华黄河坛等景区相继向游人开放，吴忠旅游知名度和影响力迅速提升。预计全年接待游客175万人次，实现旅游收入9.5亿元，分别增长17%和20%。

（二）抓战略重点，为转变发展方式提供了动力支持。抢抓自治区打造“黄河金岸”、“黄河善谷”和实施生态移民战略机遇，创造性地开展工作，拓展了新的发展空间，培育了新的发展动力。

　　打造黄河金岸增动力。切实发挥城乡建设拉动经济增长的突出作用，开工城乡建设项目273项，建筑总面积1100万平方米，完成投资180亿元，城市化率达到47%，城乡建设规模实现大幅提升。实施沿黄城市带建设项目157项，完成投资72亿元。黄河城市过境段砌护、黄河体育运动公园基本建成，京藏高速公路吴忠出口迁建工程投入使用，历史人物园启动建设。新建恒大名都、黄河外滩等57个房地产项目，建设面积430万平方米，完成开发投资26.7亿元，分别增长14%和30%。城市东南部开发建设全面加快，完成投资70亿元。新建、改造城乡公交站台275个。新建“塞上农民新居”示范点9个，整治旧村庄75个，改造危房2.1万户。张家滩湖、大坝中庄等沿黄特色小镇开工建设。新建改建农村公路765公里。同心至沿川子高速公路、国道211线灵武至甜水堡及古窑子至金积段高速公路竣工通车，全市境内高等级公路通车总里程突破1000公里。红寺堡城市道路建设及生态绿化顺利推进。盐池县城西区和城南新区开发，同心县旧城美化亮化及新区开发等大县城建设重点项目成效显著。

　　打造黄河善谷聚活力。率先在全区规划建设宁夏弘德、立德慈善工业园，出台慈善产业“十大优惠政策”，引进中烟公司、神华集团、中石油、中石化等企业集团投资项目14个，总投资94.7亿元。成立慈善基金会，首批募集善款3000多万元。争取中国长江三峡集团投资3000多万元援建宁夏“黄河善谷”残疾人职业教育培训中心，填补了全区空白。积极开展吴忠“首善”、十大“慈善之星”、百名“慈善人物”评选活动。高水平、高规格成功举办“长河大爱”慈善产业主题晚会和慈善产业发展研讨会，编辑出版了全国第一部慈善产业理论文集。人民日报、中央电视台等中央媒体对宁夏“黄河善谷”吴忠慈善产业创新做法进行聚焦报道，造血式慈善理念得到广泛宣传和认可，极大地提升了吴忠知名度和美誉度。

推进生态移民提民力。坚持生态移民与劳务移民、产业移民、教育移民相结合，共开工建设移民安置点8个，完成投资5.6亿元，建设移民住房5112套。开工建设产业项目5个，开发整理土地2.2万亩，兴修村级道路115公里，铺设人饮供水管道216公里，植树造林1690亩。移民安置区学校、村级活动场所、医疗卫生服务室、农资超市等基础设施全面配套，确保移民群众在新家园能够安居乐业、发展致富。

（三）抓生态治理，为转变发展方式提供了环境空间。扎实推进生态建设，完成营造林40万亩，新增湿地3000亩、城市绿化1.5万亩。全市城市绿地率、绿化覆盖率和人均公共绿地面积分别达到32.8%、37.4%和13.7平方米。市区创建“国家园林城市”工作顺利通过国家住建部专家组评审。完成19个农村环境综合整治项目和2个生态村镇示范项目，创建自治区级环境优美乡镇3个、生态村4个。严厉制止偷牧行为，生态恢复成果得到巩固。全市新增沼气用户8300户，总数达到6万户。新增太阳能灶1.76万台。农作物秸秆综合利用率达到53%。严格执行节能减排规定，围绕“十大节能工程”实施10项重点节能项目，对8家铁合金、铅冶炼企业落后产能进行淘汰关闭和技术更新，预计万元GDP能耗同比下降3.31%，完成自治区下达的主要污染物减排任务。

（四）抓民生改善，为转变发展方式提供了和谐氛围。高度重视保障和改善民生，全市公共财政投入民生和社会事业领域的资金达到85.5亿元，占一般预算支出的82.5%。承诺为民办理的10件实事全面完成。认真落实社会保障各项惠民政策，为2.3万名被征地农民发放养老保险补贴9200万元，解决了3.7万名企业职工基本养老保险和6千余名职工基本医疗保险遗留问题。全市5个县（市、区）全部纳入国家新农保试点，基本实现老有所养。新农合和新型农村养老保险参保率分别达到98.8%和72.7%。发放全民创业小额担保贷款2.1亿元。建设各类保障性住房7970套，发放廉租房补贴1365万元。建成居家养老服务站12个。实施同心西部安全饮水等9项农村安全供水工程，受益群众12.3万人。稳定物价工作取得积极成效，市财政安排750万元专项资金平抑市区食品类价格，新建社区蔬菜直销店50家。落实教育优先发展战略，投资2.4亿元，实施校安工程项目45个。红寺堡基本普及高中阶段教育通过自治区评估验收，同心县完成教育强县创建工作。全市高考录取率73.1%，比上年提高3个百分点。高度重视学生乘车安全问题，在全区率先实施农村学生道路交通安全保障专线工程，偏远地区中小学生实现公交车集中接送。语言文字工作率先在全区四个国家二类城市中通过自治区验收。医药卫生体制改革稳步推进，国家基本药物制度实现全覆盖并实行药品零差率销售。市人民医院通过三级乙等医院评审定级，与宁夏医科大学合作兴办回医中医院。青铜峡人民医院等5所医院迁建、18所乡镇卫生院改扩建项目顺利推进，群众就医条件得到改善。进一步深化“阳光计生行动”，人口自然增长率控制在9.17‰以内。繁荣文化体育事业取得新进步，创作完成大型歌舞剧《曼苏尔》。广泛开展“滨河大舞台”广场文化活动，农家书屋实现行政村全覆盖。市文化馆被评为国家一级馆。大力推进全民健身计划，成功举办第二届宁夏“黄河金岸”国际马拉松赛。

（五）抓民主法制，为转变发展方式提供了稳定保障。高度重视并自觉接受人大法律监督和政协民主监督。办理人大代表议案建议70件，政协委员提案280件，取得良好效果。深入推进依法行政，全面开展进一步营造风清气正发展环境活动。积极研究制定加强和创新社会管理的相关意见及配套管理办法。深入开展矛盾纠纷排查调处工作，严厉打击各类刑事犯罪，人民群众的安全感和对社会治安的满意度继续提高。大力开展学习宣传李潇、纳振东见义勇为先进事迹活动，得到全国见义勇为基金会的肯定。深入开展“小金库”、公务用车专项治理工作，加大政府投资项目审计监督力度。顺利通过国家土地督察西安局土地例行督察，国土资源管理依法行政受到国土资源部表彰。加大对宗教领域矛盾纠纷调处力度，“和谐寺观教堂”创建工作稳步推进，民族团结、宗教和顺局面得到进一步巩固。高度重视食品药品安全，集中开展严厉打击非法添加非食用物质和滥用食品添加剂、“瘦肉精”、“地沟油”等专项整治。国防动员、军民共建和双拥工作取得新成绩，我市在四年前首创取得成功的基础上，连创“全国双拥模范城”顺利通过民政部验收。安全生产、应急管理和防震减灾工作不断加强，人防、档案、外事、统计、住房公积金、妇女儿童等工作取得新成效。

各位代表！这一年，考验前所未有，收获提振信心。我们深深体会到：科学发展是跨越发展的重要前提，必须长期坚持。坚持把保持经济平稳较快增长作为首要任务，以加快转变发展方式、调整优化产业结构为主线，以实现城乡统筹、山川共济和经济社会协调发展为目标，采取一系列科学应对的有效举措，克服一系列超乎寻常的困难矛盾，才能取得一系列令人振奋的发展成就。创新驱动是跨越发展的不竭动力，必须持之以恒。自治区打造“黄河善谷”战略构想是落后地区摆脱困境、化劣势为优势的思维创新，慈善产业蓬勃兴起是吴忠人民抢抓先机、变输血为造血的实践开拓。慈善产业的创新突破让吴忠为世人关注，汇聚了关怀、得到了支持、吸引了投资，为经济社会发展注入了活力、增添了后劲。干部群众的思想观念发生了转变，劲头更大、信心更足。县域经济是跨越发展的重要支撑，必须常抓不懈。把做大做强县域经济作为重中之重，采取提高以奖代补幅度、完善考核评比制度、强化督查落实力度等有效举措，调动了县域经济的发展活力，激发了县域经济的竞争魄力，增强了县域经济的创新能力，比学赶超、争先创优的县域经济强势发展为全市经济社会发展提供了坚实支撑。凝心聚力是跨越发展的重要保证，必须始终发扬。我们牢牢把握各族人民群众的期待和愿望，紧紧依靠各族人民群众的智慧和力量，珍视团结，弘扬友善，倡导包容，发展思路得到了群众支持，城乡变化换来了群众认可，惠民举措赢得了群众拥护。广大干部群众在面对困难和挑战时同心同德、同甘共苦，以实际行动巩固和发展了科学发展的好势头、和谐稳定的好局面。

各位代表！回顾过去一年的工作，能够顶住各种压力实现逆势突破，这是全市上下始终坚持正确方向、谋求广泛支持、凝聚各方力量所取得的重大胜利。成绩得益于自治区党委、政府的坚强领导，得益于市委的正确决策，得益于人大、政协的鼎力支持，得益于社会各界的共同努力。在此，我代表市人民政府，向全市广大工人、农民、知识分子、干部职工，向人大代表、政协委员、民主党派、工商联、人民团体、离退休老同志和社会各界人士，向驻吴人民解放军、武警和消防官兵、政法干警，向中央及区属驻吴单位，表示崇高的敬意和诚挚的感谢！

肯定成绩的同时，我们也清醒地看到，困扰发展的矛盾和问题仍然突出。主要是：经济总量小、发展基础弱，综合竞争力还不强的局面没有从根本上改变；工业主导作用发挥还不明显，现代农业发展还不快，服务业层次还比较低，经济增长的质量和效益还不理想，节能减排、环境保护、安全生产的压力还比较大；群众关注的住房、物价、就业、教育、医疗等很多实际问题解决的还不够理想，民生改善力度与群众期待还有一定差距；政府自身建设仍然存在一些薄弱环节，不思进取、脱离群众、作风漂浮、效能低下的现象还不同程度存在。对于这些，我们将在今后的工作中切实加以解决，努力把各方面工作做得更好。

继续砥砺奋进的2012年

今年是全面贯彻落实吴忠市第四次党代会精神的第一年。继续巩固当前发展势头，顺利推进“十二五”规划实施，是时代赋予我们的历史重任。

根据市第四次党代会和四届二次全委会议精神，今年政府工作的总体要求是：高举中国特色社会主义伟大旗帜，以邓小平理论和“三个代表”重要思想为指导，全面贯彻党的十七大、十七届三中、四中、五中、六中全会精神，以科学发展、跨越发展为主题，以加快转变发展方式为主线，以建设宁夏沿黄经济崛起区、生态文明示范区、城乡统筹发展样板区、民族团结进步模范区、黄河善谷先行区为目标，坚持做大经济总量与提高质量效益并重，坚持城乡统筹与山川共济并重，坚持保障改善民生与创新社会管理并重，坚持生态建设与环境保护并重，全面加快经济建设、文化建设、社会建设、生态文明建设和政府自身建设，努力确保超额完成本届政府各项目标任务，为实现“十二五”目标迈出更加坚实的一步。

主要预期目标和约束性指标是：地区生产总值增长12%以上；地方财政一般预算收入增长20%以上；固定资产投资增长30%以上；社会消费品零售总额增长18%以上；城镇居民人均可支配收入增长10%以上；农民人均纯收入增长10%以上；人口自然增长率控制在10‰以内；城镇登记失业率控制在4%以内；完成自治区下达的年度节能减排任务和控制物价目标。

各位代表！实现今年各项目标任务，我们必须科学研判发展形势，切实增强工作的预见性、针对性和有效性，努力推动全市经济社会发展再上一个新台阶。

准确把握“一个基调”，应对新挑战。今年，国内外经济环境更为严峻复杂，宏观经济下行压力明显加大，我市经济运行的不确定性和不稳定性上升，经济社会发展面临新的更多挑战。中央确定的经济工作总基调是“稳中求进”。准确把握这个总基调，必须结合吴忠实际，认清发展不足是我市最大的市情。必须把实现经济增长目标作为首要任务，把做大经济总量作为当务之急，把保持又好又快作为坚定方向，善于在变局中把握机遇，在逆境中主动作为，在挑战中勇于胜出，以快的增长速度保障跨越需求，以大的经济增量促进结构优化，以好的发展质量应对困难挑战。

紧紧盯住“两大任务”，抢抓新机遇。主动对接争取中央实施西部大开发战略、扩大内需政策、发展实体经济、保障和改善民生、启动新一轮扶贫攻坚计划等战略举措所蕴含的“政策机遇”。准确把握争取国家推进宁夏沿黄经济区、蒙陕甘宁能源“金三角”、振兴陕甘宁革命老区等区域规划所带来的“规划红利”。积极参与争取自治区打造向西开放前沿阵地、发展内陆开放型经济等对外开放战略所创造的“开放动力”。把“两大任务”作为突出抓手，加大力度向上争取更好的政策和更大的支持，想尽办法引进更好的项目和更大的投资，力争全年上争资金和招商引资分别突破100亿元和400亿元，再上一个大台阶，为经济社会发展提供源源不断的动力。

着力突出“三大重点”，再造新优势。打造“黄河金岸”、“黄河善谷”、实施生态移民是自治区党委、政府的战略部署，也是事关我市发展全局、必须全力突破的三大重点。我们要发挥好打造“黄河金岸”的独特优势，全面加快功能优化、品位提升和规模扩张，着力构建宜商、宜居、宜业的环境优势。抢抓打造“黄河善谷”先行先试机遇，做好慈善兴业文章，营造慈善文化氛围，着力构建具有产业吸引力和道德感召力的发展优势。探索实施生态移民、劳务移民、教育移民的新机制，切实把移民工作、扶贫开发与产业发展、技能培训同规划、同实施，提升移民及农村转移劳动力的就业创业兴业能力，着力构建人力资源优势。

切实提升“四种能力”，提供新保障。提升财政保障能力，加强税收征管，努力壮大一批企业、建设一批项目、培植一批税源，确保公共财政收入实现预期增长，为保发展、保民生、保重点提供保障。提升融资服务能力，充分调动政府、银行、企业三个方面的积极性，有效整合公共担保平台、金融信贷支持、民间资本运作三个渠道的力量，帮助企业特别是中小企业、微型企业解决资金难题，为发展实体经济提供保障。提升运行调度能力，建立完善政府经济运行监测研判和跟进服务机制，有针对性地定期开展经济运行调度，积极协调帮助企业解决煤、电、油、运等困难，及时解决群众关心的物价、市场供应、食品安全等生产生活问题，为稳固经济运行质量提供保障。提升维护稳定能力，始终坚持把维护稳定作为压倒一切的责任，认真落实加强社会管理创新各项举措，主动解决民生领域的困难和矛盾，妥善处理各类突发公共事件，注重从源头上预防和减少不稳定因素，为聚精会神搞建设、一心一意谋发展提供保障。

努力开创各项工作新局面

科学推进今年的各项工作，必须紧紧依靠和团结全市各族人民，继续以大举措克服大困难，以大手笔推动大建设，以大气魄谋求大发展。重点做好以下八项工作：

（一）围绕“结构”下功夫，力促新型工业再求新突破。大力推进兴工强市战略，以调整优化产业结构为主线，全面加强对工业经济的扶持推进力度，确保规模以上工业增加值增长16%，突破百亿元大关。加大项目建设力度。力争年内新建、续建工业项目120个，完成固定资产投资130亿元。重点加快通达100万吨煤化工、合力汽车专用车制造等38个在建项目建设进度，力促深然众源公司100万立方天然气液化等82个新上项目开工建设，金风煤矿等62个建成项目达产达效。力争全年新增规模以上工业增加值15亿元以上。加快结构调整升级。重点支持发展能源化工、新材料、装备制造、食品加工四大优势产业，使之逐步成为百亿元产值的战略性产业。年内，全市风电、光电总装机容量分别达到90万千瓦和20万千瓦，食品加工业实现产值130亿元。鼓励和支持建材、冶金等传统产业加快技术改造升级，实施千万元以上技改项目15个，提升传统产业的抗风险能力和市场竞争力。大力扶持企业成长。认真组织实施自治区“百家成长、千家培育”扶持计划，做强大型企业，做大中型企业，做多小型微型企业。全力扶持万盛生物等13家中型企业成长为自治区级骨干企业，帮助杞叶青生物饮料等150家中小企业加快发展。发挥各类中小企业孵化基地的硬件优势，力争年内新入园中小企业80家以上。加大上争资金力度，落实自治区“五优一新”等中小企业扶持资金700万元。发挥各类中小企业融资担保平台作用，发放各类企业扶持贷款不低于2亿元。强化园区支撑作用。市财政安排4000万元工业专项资金，支持工业园区基础设施建设和重点项目。强化市级统筹，实施分类指导，优化项目布局，形成各具特色、各具优势的发展格局。充分利用牛首山工业园、青铜峡新材料基地等园区资源，实施灵活的管理体制和招商政策，大力推进工业项目上山入园步伐，促进土地集约节约利用。完成工业园区基础设施投资4.5亿元，落实招商引资项目资金180亿元。

（二）围绕“规模”下功夫，力促现代农业再上新台阶。把发展特色农业、高端农业和节水农业作为主攻方向，加快推进现代农业发展，促进农业增效、农民增收。全年完成农业总产值89亿元，增长5%。推进特色产业规模化。加快“十大基地”建设，优质粮食种植面积稳定在100万亩，新增设施农业1万亩、高酸苹果1.1万亩、葡萄3万亩、人工甘草5万亩、红枣2.3万亩、枸杞3万亩。新增奶牛3万头、肉牛5万头、羊20万只。新建市级以上现代农业示范基地40个，力争全市规模化奶牛示范牧场达到30个。加快孙家滩现代农业示范区建设，打造宁夏中部干旱带现代农业核心示范区。推进优势产业品牌化。围绕优势特色产业，加快引进和培育农业产业化龙头企业。重点支持伊利乳业、中航塞外香、冠丰种业、天予枣业等产业化龙头企业扩规模、打品牌，提升市场竞争力。新培育示范合作社10个，提高农业社会化服务程度。加强产销对接、场店对接、农超对接，加快无公害、绿色、有机农产品示范基地建设及认证工作，提高市场占有率。推进技术应用普及化。大力普及农业新品种、新技术、新装备，加快推进农作物良种、畜禽改良和农业新技术推广应用，力争农作物良种覆盖率、畜禽良种改良率分别达到95%和98%以上，进一步提高科技服务农业的水平。建立动物防疫、农产品质量安全及农业风险防范体系，提高农业安全保障能力。推进基础建设标准化。抢抓国家加快水利改革发展的重大机遇，继续大力开展农田水利基本建设。实施苦水河防洪工程、小农水重点县、小流域综合治理等一批水利工程。完成农田整治80万亩，改善灌溉面积20万亩，新增节水灌溉面积15万亩，发展高效节水补灌面积10万亩，治理水土流失面积240平方公里。实施利通区金积、高闸和红寺堡鲁家窑移民区等民生水利工程，解决3.5万人安全饮水问题。

（三）围绕“提升”下功夫，力促现代服务业再有新进展。把发展现代服务业放在更加突出的位置，大力发展以商贸物流业、旅游业、特色餐饮业为龙头的现代服务业，打造全区乃至西部具有较强辐射力的区域性商贸物流中心、生态旅游文化中心和休闲娱乐度假中心。做强商贸物流业。加快推进利通区“十大市场”、“三大汽车服务区”、废旧物资回收市场建设，力促金属材料物流园等市场建成投入运营。大力实施红寺堡移民区市场、盐池蔬菜批发市场、同心永昌建材城等专业市场建设，加快构建以利通区为中心的现代物流业发展格局。加强农村商贸流通和市场体系建设，继续实施“万村千乡市场工程”，改造提升农家店30个，推动现代物流业态向农村延伸，大力拓展农村消费市场。做大文化旅游产业。认真贯彻自治区《关于做强做大文化旅游产业的决定》，加强文化与旅游的互动融合，重点发展以黄河文化游、红色经典游、绿色生态游和近郊休闲游为主的特色旅游经济。加快完善中华黄河坛配套设施建设，完成黄河楼、历史人物园建设，启动实施黄河宁夏吴忠段航运工程项目一期工程航道整治，打造“夜游黄河”独特品牌，构建黄河文化旅游精品线路。举办“黄河金岸”文化旅游节。力争全年实现接待游客200万人次，旅游收入突破10亿元大关。促进房地产及新型服务业健康发展。继续把房地产业作为拉动投资、促进消费的重要产业，促进房地产业稳定健康发展，确保商品房销售价格保持在合理区间。加快金融保险、信息中介、电子商务等新型服务业发展。

（四）围绕“统筹”下功夫，力促城乡建设再迈新步伐。抢抓自治区打造沿黄经济区“四带一高地”战略机遇，按照“拓展强壮新区，改造提升老城，做大做活县城，整合聚集村镇，整治优化环境”思路，加快以城促乡，推进城乡一体化进程，力争全市城市化率达到49%。提升黄河金岸建设水平。高起点规划、高标准实施滨河新区中心商务区、美食购物广场、滨河五星级酒店、建设大厦、现代传媒中心等45个重点项目。完成市区经二街、纬五路等8条城市道路和城市西区集污管网续扩建、新区供水供热管网等项目。青铜峡市加快城市东区建设，实施汽车运输调度中心、东区商贸中心等基础设施建设项目。加快黄河两岸同城化步伐，推进商贸、金融、医疗、通讯、交通等居民生活必需的服务网点向黄河两岸集中。加快老城区改造步伐，抓好城市东南部道路、绿化、水系等基础设施续建工程。加强道路交通、市容市貌和社区物业管理，增加市区停车泊位，优化城市交通及公交线网布局，方便群众出行，提升城市管理水平。推进县城建设扩容提质。加快实施“大县城”战略，引导生产要素、优势资源向县城集中，形成集聚辐射功能较强的县域经济中心。着力实施好红寺堡区城市南北大门改造，盐池县石油大厦，同心县老城改造新区建设等重点项目。加快县城城市道路等基础设施建设，开工建设利通区至红寺堡快速通道。加快小城镇和新农村建设。紧抓国家实施“百县中心镇工程”建设机遇，大力推进村镇整合集聚，实施金积、韦州、惠安堡等中心集镇建设和古城湾二期、张家滩湖小镇等沿黄特色村镇建设。深入实施塞上农民新居、村庄整治和危窑危房改造工程，新建示范点7个，整治旧庄点70个。新建改建农村公路800公里，推进公交客运城乡一体化。加强生态环境建设。巩固“国家园林城市”创建成果，启动“国家环保模范城市”创建工作。全市完成营造林30万亩，新增城市绿化面积1万亩。加强生态防护林体系建设, 加大禁牧封育工作力度，继续实施退耕还林、天然林保护、防沙治沙、湿地保护等生态项目。深入推进重点区域和行业“三废”综合治理。大力开展农村环境集中连片整治工程，完善“两大工程”长效机制，巩固农村综合环境整治成果。

（五）围绕“创新”下功夫，力促慈善兴业再谱新篇章。把创新发展慈善产业作为推进慈善兴业的着力点，为实现和谐发展提供强劲动力。夯实园区发展平台。加快慈善园区基础设施建设，弘德工业园10平方公里“五通一平”和残疾人创业园标准化厂房建成投入使用，完成园区基础设施投资３亿元，提升园区项目承载能力。规划建设同德工业园，为同心县搭建新的扶贫济困产业平台。建立慈善签约项目跟踪落实机制，全力推进弘德印刷包装项目、金风嘉泽产业园、内蒙古蒙草种植等14个引进项目加快建设，力争完成投资40亿元。完善政策扶持体系。积极争取国家有关部委及自治区支持，针对慈善产业出台更为具体的供地、税收、电价等优惠政策，严格兑现承诺，支持企业加快发展。继续向国家民政部申请“中国慈善产业试验区”，力争获得更高层次、更大范围支持，把吴忠打造成为名副其实的慈善家与企业家投资兴业、扶残济困的“政策洼地”和“道德高地”。加大招善引资力度。充分利用各类经贸文化平台，策划组织举办善博会、慈善产业与慈善项目推介会等各类活动，吸引国内外慈善家、企业家及公益慈善组织到吴忠实施项目。力争年内再引进一批慈善项目落户慈善园区，合同资金100亿元。创建全国慈善城市。按照“慈善产业、社会捐赠、慈善组织、慈善项目、志愿服务、政府支持、慈善文化”七个指标体系，启动“全国慈善城市”创建活动，力争进入第二届“中国城市公益慈善指数”评选发布活动先进行列。开展残疾人免费健康体检等助残活动，实施远东残疾人创业一条街项目，加快残疾人培训基地建设，让残疾人享受更多关爱，生活更加体面、更有尊严。

（六）围绕“深化”下功夫，力促改革开放再增新动力。最大限度地增强发展动力、释放发展活力。深化重点领域和关键环节改革。巩固政府机构改革成果，以转变政府职能为重点，积极稳妥地推进事业单位分类改革。完善公共财政体系，优化财政支出结构，加强预算管理，提高财政资金使用效率。完善国民经济核算体系，全面推进企业统计改革。深化农村改革，规范和完善农村土地承包经营权流转。积极推进集约节约用地，优化土地利用结构，切实保障重大基础设施和重点产业发展用地，确保全市基本农田面积不减少，质量不降低。提高对外开放水平。充分利用中阿经贸论坛等重大活动，加强与中东国家和地区贸易、投资合作。积极扩大与周边地市的交流合作，推动与新疆吐鲁番、内蒙古阿拉善盟的深度合作。创造性地办好第二十三届中国西部商品交易会等系列活动，在促进招商引资、展示城市魅力、扩大对外宣传等方面取得实效。

（七）围绕“和谐”下功夫，力促社会民生再获新改善。集中力量加大民生投入，切实解决群众最关心、最迫切、最需要的困难和问题。稳步推进生态移民。全面完成第一批9个安置点建设，搬迁安置2.32万人，完成第二批4082套移民住房及配套设施、土地开发改造、种养殖设施建设等任务。加大扶贫开发和社会救助力度，整合各类项目和资金，重点支持贫困地区优势特色产业发展，千方百计促进贫困地区群众增产增收。促进教育均衡发展。做好改善民办教育发展环境等三项教育改革，扎实推进利通区、青铜峡义务教育均衡发展试点工作，推动教育规范、均衡发展。完成同心县基本普及高中阶段教育工作，实现全市基本普及高中阶段教育目标。继续实施校安工程，加强学校和校车安全管理。高度重视学前教育和职业教育发展。加快医药卫生体制改革。完善基层药品供应保障体系。全面推进公立医院改革，加快县级综合医院改革步伐，开工建设市人民医院和利通区人民医院。健全乡村、社区等基层医疗卫生服务体系。加强人口和计划生育工作。稳定低生育水平，提高出生人口素质，统筹解决人口问题。全面推进人口和计划生育综合改革示范市创建活动。促进文化体育事业蓬勃发展。大力培育地方特色文化产业，提升文化产业社会化运作水平，力争开工建设黄河文化产业园。完成农村卫星电视直播户户通工程。高水平举办好第三届宁夏“黄河金岸”国际全程马拉松赛等体育赛事，大力发展竞技体育，广泛开展全民健身系列活动。完善社会保障体系。深化国家创业型城市创建工作，认真落实各项创业促就业政策。深入推进社会保障体系建设，全面启动统筹城乡居民养老保险、医疗保险工作。实施社会保障“一卡通”工程，推行居民医保大病门诊统筹和普通门诊统筹，扩大保障范围，提高保障水平。完善失地农民社会保险制度，做好失地农民就业与生活保障工作。新建保障性住房11510套，确保建设质量和分配公平。利通区率先在全区实现城市低收入家庭廉租房实物配租保障全覆盖。认真做好承诺为民办理的10件实事。做好物价稳控工作。建立粮食安全应急保障体系，着力保障重要农副产品的生产和供应。新建蔬菜直销店20个，开设早晚市场7个，适时启动困难群众动态生活价格临时补贴机制，优先保障困难群众吃到平价的优质粮、新鲜菜、放心肉。

（八）围绕“稳定”下功夫，力促社会管理再创新局面。深入研究社会管理问题，准确把握社会管理规律，认真落实社会管理政策措施，构建更加科学的社会管理体系。加强民族宗教工作。依法加强和创新宗教事务管理，实行领导干部联系宗教人士制度，充分发挥宗教团体和宗教界人士在普法宣传、反映民意、化解矛盾、维护团结方面的作用，着力提高宗教事务依法管理水平。深入推进民族团结进步模范市创建工作。加强社会综合管理。全面开展“六五”普法，增强社会法律意识和法制观念。加强社区阵地建设，将社区办公用房和居民公益性服务设施纳入城市建设总体规划。建立和完善群众利益诉求表达机制和社会矛盾调处机制，使矛盾纠纷化解在基层，解决在萌芽状态。畅通信访渠道，规范信访秩序，妥善解决群众反映强烈的突出问题，积极预防和妥善处理群体性事件。继续完善流动人口、刑释解教人员等人群的管理服务工作。加强安全小区建设，优化社区治安防范体系。强化网络舆论引导，加强互联网等新兴媒体管理。整合现有网络资源，建立以吴忠市门户网站为主站的网站体系。加强食品药品监管，严厉打击各种制假、售假违法活动，坚决让伪劣食品药品在吴忠没有制售的土壤和市场。严格安全生产责任制，切实抓好较大安全事故隐患的排查和整改，杜绝重大安全生产事故发生，加强交通安全监管力度，提升人民群众安全生活幸福指数。加强精神文明建设。强化社会主义核心价值观体系建设，深入推进社会公德、职业道德、家庭美德、个人品德建设，不断拓展群众性精神文明创建活动。抓好国防教育、国防动员和民兵预备役工作，提高双拥共建水平。支持工会、共青团、妇联、科协等人民团体工作，进一步增强共谋发展、共促和谐的工作合力。

切实加强政府自身建设

各位代表！本届政府四年来，深受各级各界关怀和支持。我们始终坚持在完善自身建设中谋划新发展，在干事创业中树立新形象，尽心竭力、不敢怠慢。今年是本届政府依法履职的第五年，更是冲刺之年。做好今年的政府工作，确保各项奋斗目标顺利实现，必须把营造风清气正发展环境作为着力点和切入点，力求在以下五个方面有大改观、新突破。

　　大力加强学习调研。选择学习就是选择发展，坚持学习就是坚持进步。引导政府公务人员牢固树立终身学习理念，大力弘扬浓厚的学风、调查风、研究风，吃透上情、掌握市情、了解民情，切实提高各级公务人员的学习本领、工作本领和创新本领。认真落实人才队伍建设规划，有计划、有针对、有系统地开展多种形式的培训教育活动，让政府公务人员更多地把视野转向先进理念，把心思用在开拓创新，把身子沉到基层，达到学以明理、学以养德、学以开智、学以增勇、学以促廉的效果。

继续优化政务环境。启动市级政务大厅改扩建项目，新建吴忠市政务服务中心，将市直机关所有行政审批事项和收费事项全部纳入服务中心集中公开办理。健全完善四级政务服务网络，推进政务服务中心标准化建设，积极推行网上审批。牢固树立“企业创造财富、政府创造环境”的理念，自觉寓管理于服务之中，切实做到亲商、爱商、扶商。对投诉的各类服务问题，一经核实坚决问责。强化对金融机构的支持，为政银企合作创造宽松条件，为优化金融环境做好服务和保障。

坚决做到依法行政。各级行政机关要自觉在宪法和法律范围内活动，严格依照法定权限和程序履行职责，切实提高行政行为的公正性、合法性和公开透明性。依法开展行政复议和应诉工作，努力预防和化解行政争议。自觉接受人大法律监督和政协民主监督，高度重视司法监督、舆论监督和社会公众监督。认真听取民主党派、工商联、无党派人士和各人民团体的意见，深入推进政府决策科学化、民主化、法治化。

不断提升行政效能。进一步健全部门协调配合机制，形成工作合力。强化效能意识，完善督查机制，加强跟踪落实和监督检查，坚决杜绝有令不行、有禁不止的现象，确保政令畅通。不断提高真抓实干、开拓创新、促进发展的能力，确保政府承诺的事情、定下的工作坚决落实到位。扎实开展“庸、懒、散”专项治理，重点解决一些单位人心不齐、士气不振、执行不力，一些干部不谋其职、不思进取、不敢负责等问题。

扎实推进廉政建设。认真落实党风廉政建设责任制，进一步完善土地出让、政府采购、工程建设招投标等制度，坚决堵住一些重要部门、核心岗位、关键环节权力运行的监管漏洞。强化领导干部经济责任审计，严厉查处各类违法违纪行为，坚决惩治腐败现象。深入开展政风行风评议活动，对群众反映强烈的问题实行挂牌督办。严格控制“三公”支出，把有限的资金更多、更好地用于促进发展、改善民生。

各位代表！

无论形势多么复杂多变，道路多么崎岖艰辛，任务多么纷繁沉重，勇于担当使命、忠实履行职责、全力完成任务是我们的一贯承诺和坚定追求。让我们高举中国特色社会主义伟大旗帜，紧密团结在以胡锦涛同志为总书记的党中央周围，在市委的坚强领导下，全面贯彻落实科学发展观，同心协力，开拓创新，锐意进取，扎实工作，以优异成绩迎接党的十八大胜利召开，为建设和谐富裕新吴忠而努力奋斗！
